# Supplementary figures and images for: A novel male accessory gland peptide reduces female post-mating receptivity in the brown planthopper
Source: PLoS Genet. 2025 May 6;21(5):e1011699. doi: 10.1371/journal.pgen.1011699 (PMC12077777; doi:10.1371/journal.pgen.1011699)

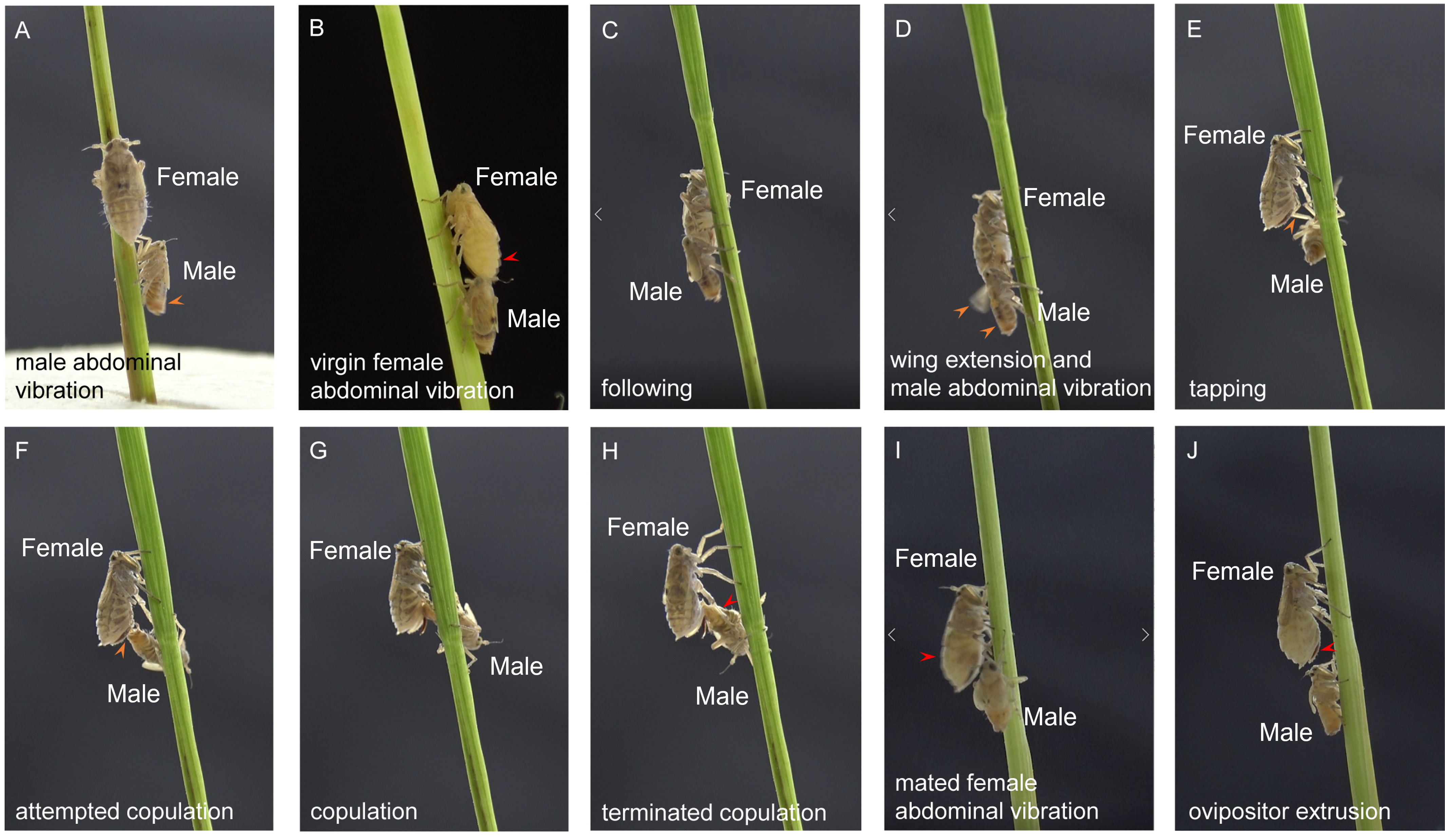

Supplement: S1 Fig — A-H: The mating behavior sequence. The sequence includes eight steps (A-H, following, wing extension, abdominal vibration, abdominal rubbing, attempted copulation, copulation, terminated copulation and leaving). The larger individual is the female and the smaller is the male. I and J: The post-mating response behaviors. (TIF) [file pgen.1011699.s009.tif]

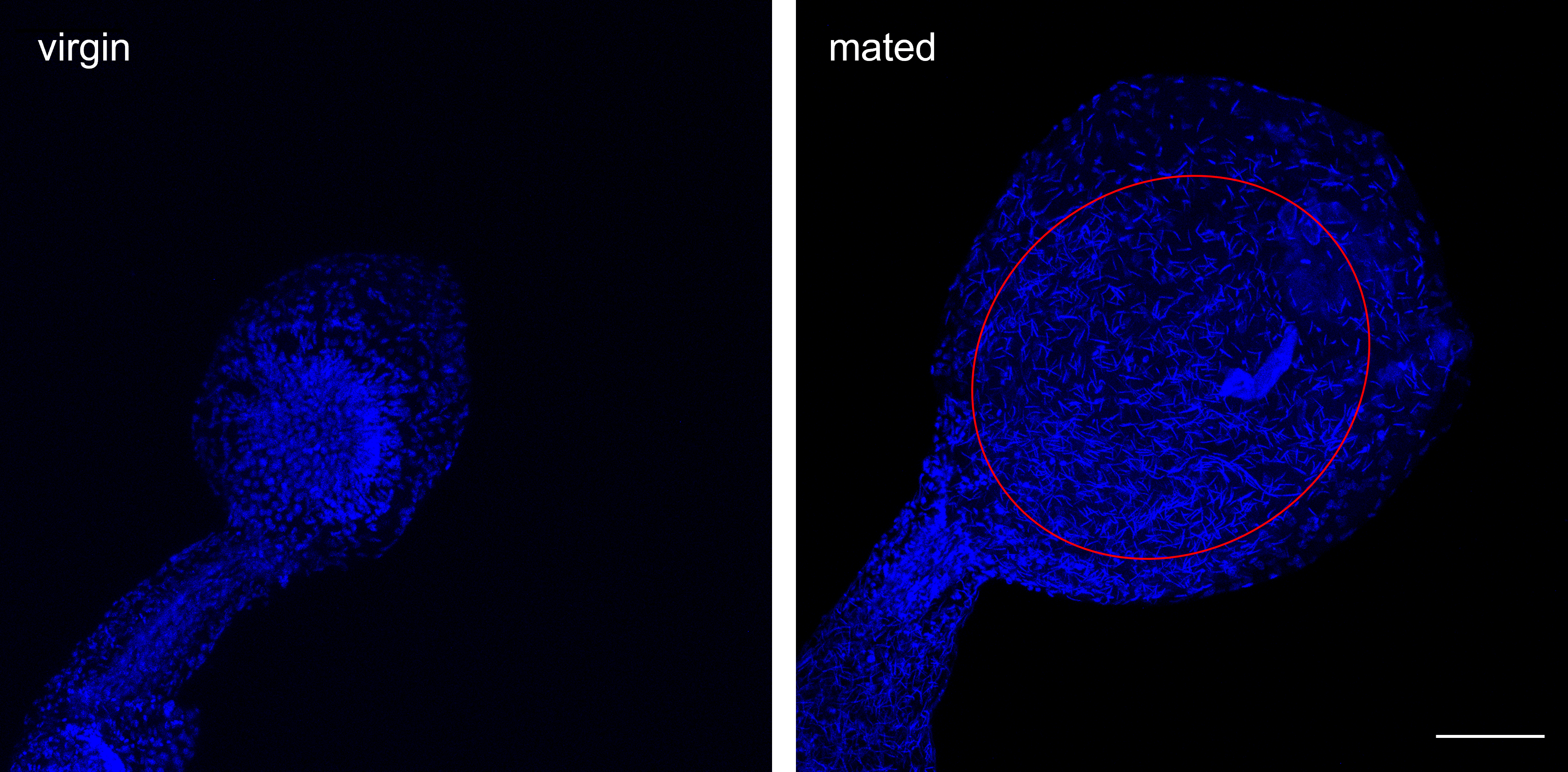

Supplement: S2 Fig — The sperm were stained with DAPI (red circle in the mated female). Scale bar: 100 μm. (TIF) [file pgen.1011699.s010.tif]

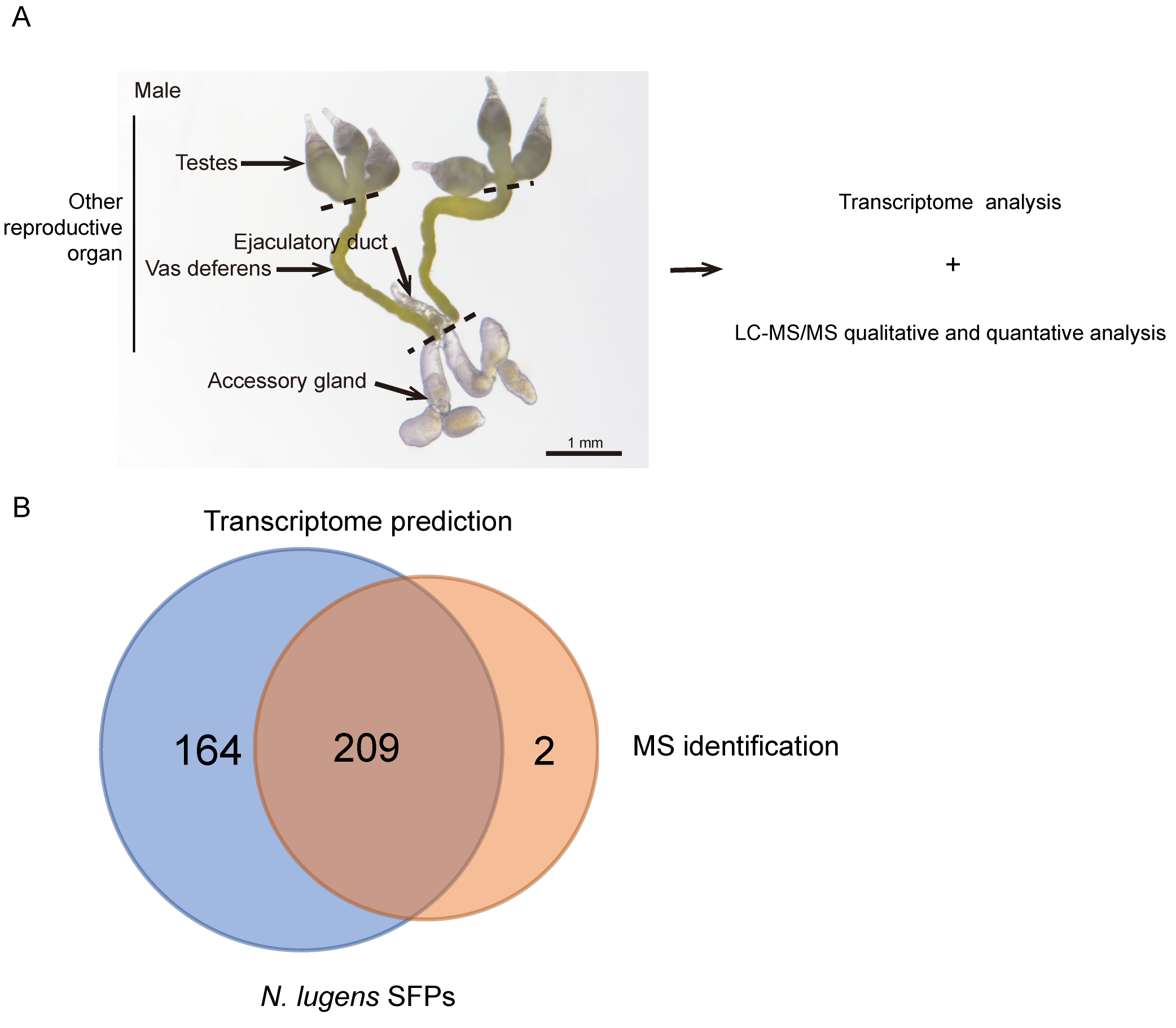

Supplement: S3 Fig — Dissected accessory glands were used for extraction and subjected to transcriptome and proteome analysis using liquid chromatography (LC) and mass spectrometry (MS/MS). (B) Venn diagram of the numbers of predicted seminal fluid proteins comparing transcriptome prediction and MS identification. (TIF) [file pgen.1011699.s011.tif]

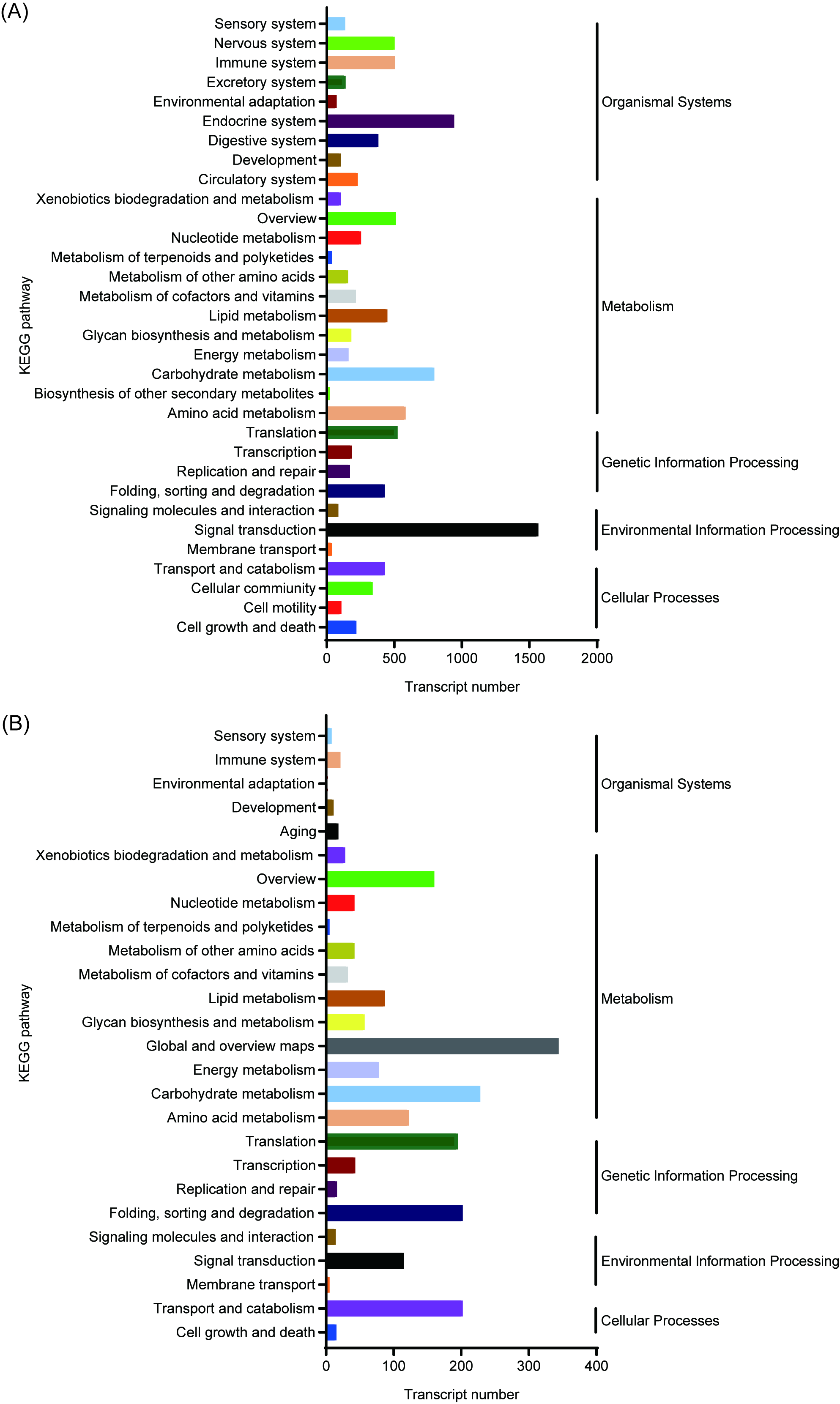

Supplement: S4 Fig — (A) Classification based on transcript. (B) Classification based on protein. (TIF) [file pgen.1011699.s012.tif]

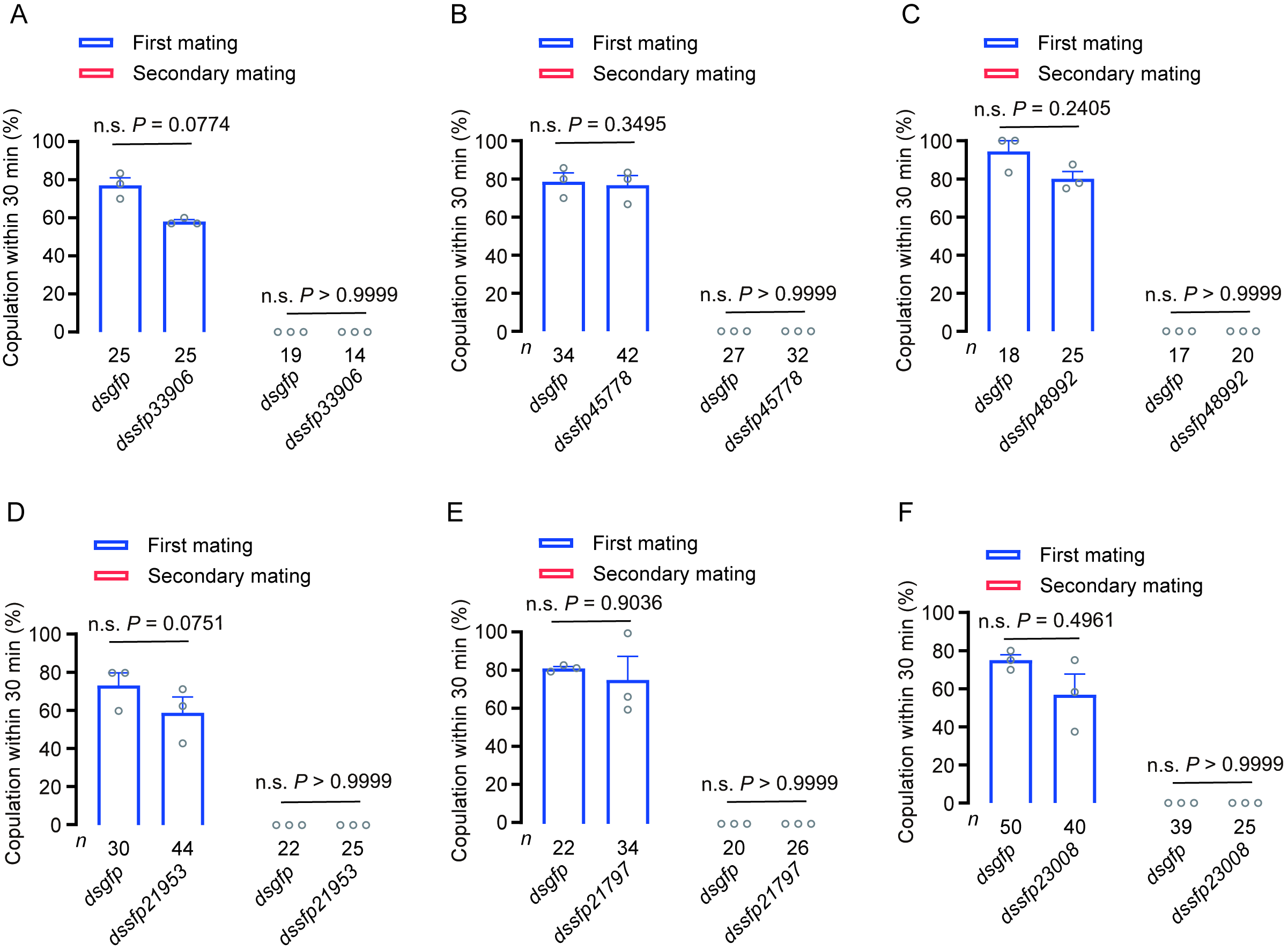

Supplement: S5 Fig — The small circles denote the number of replicates; the numbers below the bars denote total number of animals. Data are shown as mean ± s.e.m. *P < 0.01, and ns (non-significant), P > 0.05, two-way repeated measures ANOVA followed by šídák’s multiple comparisons test. The annotations of these seminal fluid genes were showed below.Sfp33906: seminal fluid protein (APA33906.1). Sfp45778: melanization protease 1-like. Sfp48992: N(3)-methylcytidine methyltransferase METTL6. Sfp21953: GTP-binding nuclear protein Ran. Sfp21797: seminal fluid protein (APA33927.1). Sfp23008: seminal fluid protein (APA33928.1). (TIF) [file pgen.1011699.s013.tif]

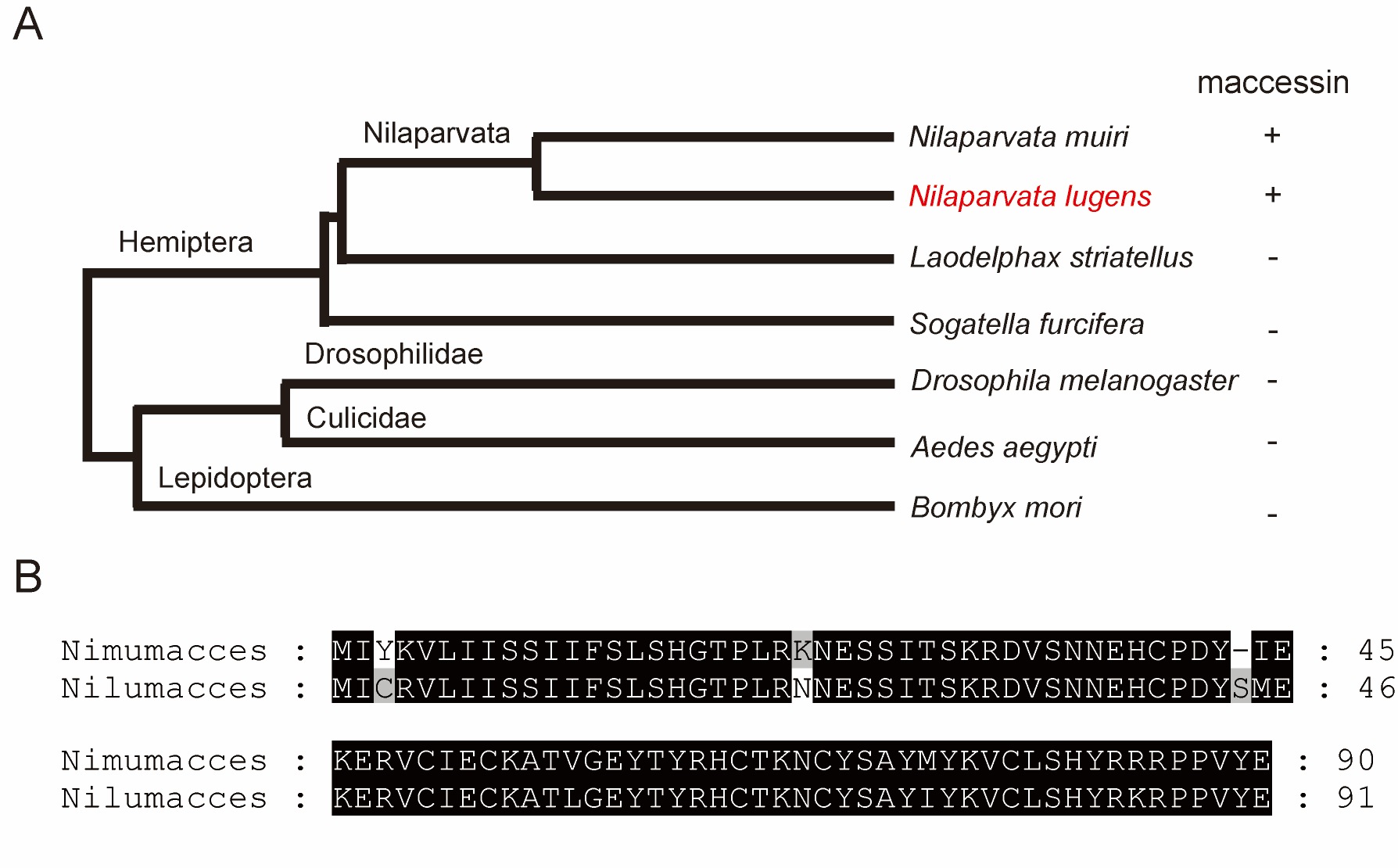

Supplement: S6 Fig — (A) The presence of maccessin in different species. (B) The alignment of maccessin protein sequence between Nilaparvata muiri (upper) and Nilaparvata lugens (lower). (TIF) [file pgen.1011699.s014.tif]

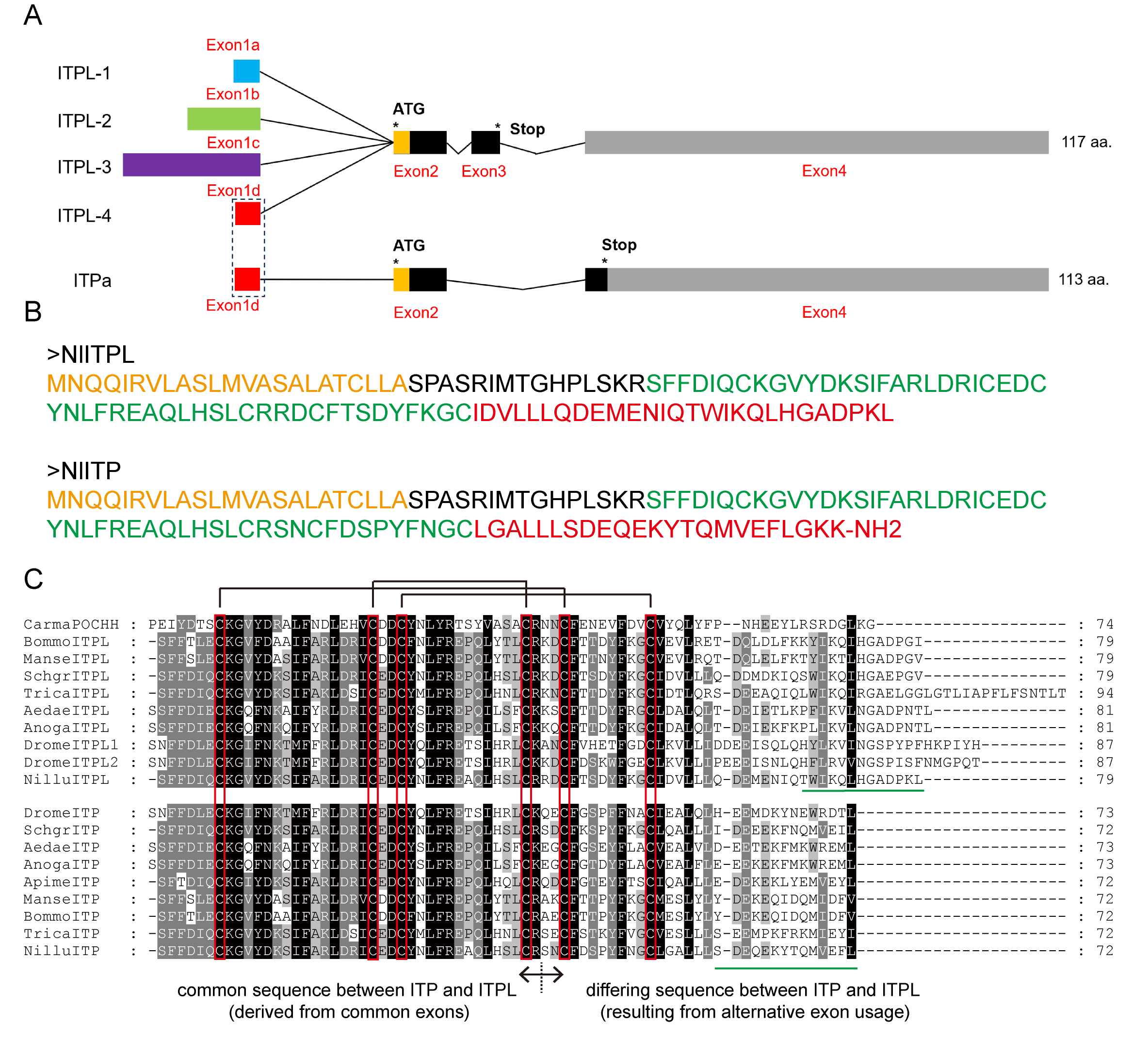

Supplement: S7 Fig — A The identified ion transport peptide/ ITP-like (ITPa/ITPL) transcripts in the brown planthopper. The coloured blocks represent exons within the ITP/ITPL transcripts. Exons 1a, 1b, 1c and 1d are alternative 5’ untranslated regions used by ITP and ITPLs. * denote the start codon and stop codons of the transcripts. B. Amino acid sequence of ITP and ITPL of the brown planthopper. Orange indicates sequence of signal peptide; green indicates mature peptide sequence; red indicates difference sequence of ITP and ITPL. Note that four slice forms of itpl are known (itpl-1–4), which all could give rise to the same mature ITPL peptide. C. Multiple comparisons of ITP and ITPL mature peptides in brown planthopper and other species. The red frames indicate conserved cysteines. Deduced ITP and ITPL sequences are shown for Manduca sexta (Manse, AY950500, AY950501), Bombyx mori (Bommo, AY950502, AY950503), Schistocerca gregaria (Schgr, XP_049859893.1), Apis mellifera (Apime, XP_006571870.1), Aedes aegypti (Aedae, AY950504, AY950505, AY950506), Anopheles gambiae (Anoga, EAA09451.4, XP_061497799.1), Drosophila melanogaster (Drome, NP_001036569.2, ABZ88141.1, NP_001163293.1), and Tribolium castaneum (Trica, EFA07585). (TIF) [file pgen.1011699.s015.tif]

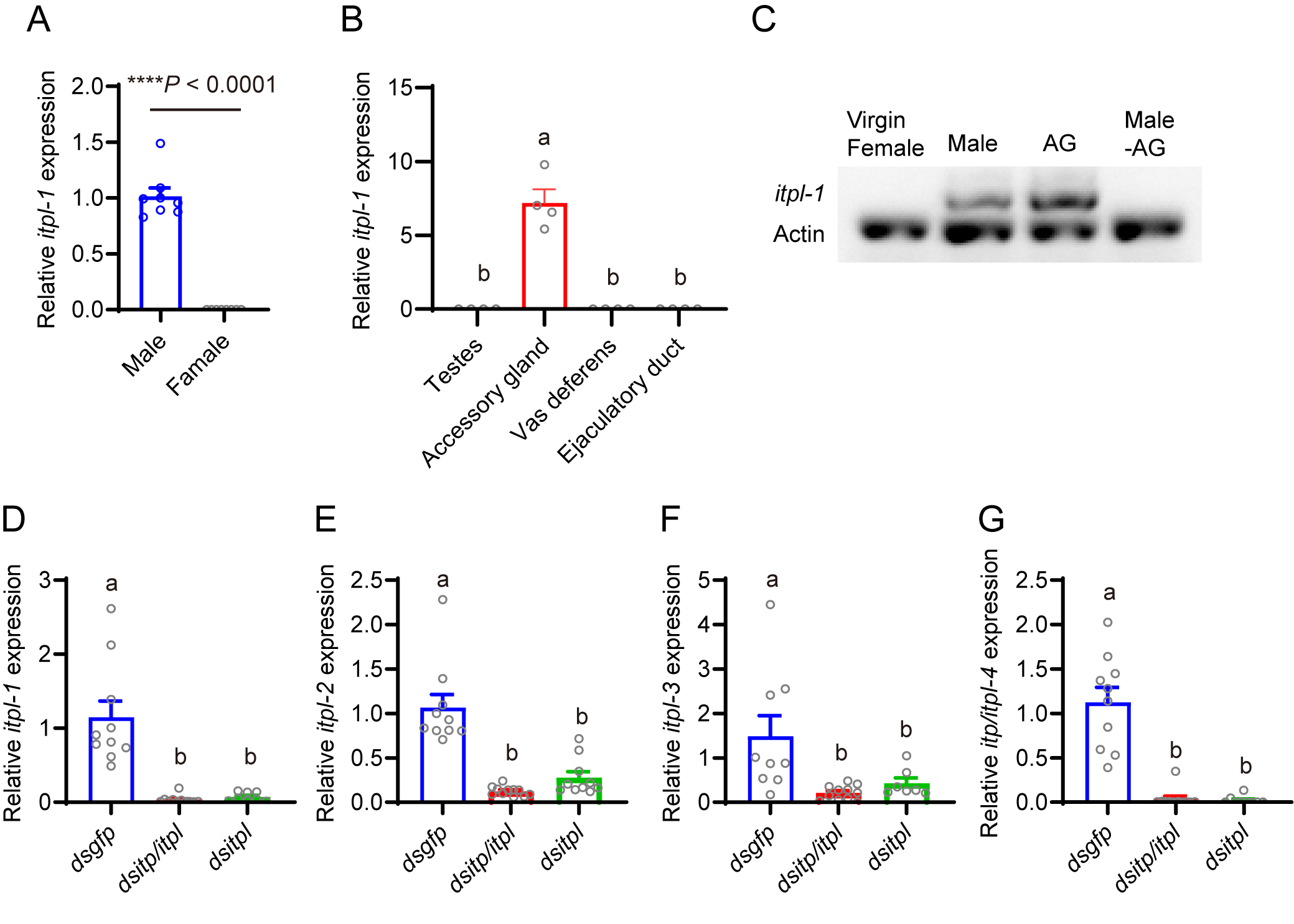

Supplement: S8 Fig — A. Relative expression of itpl-1 gene in N. lugens at different sexes. Data are shown as mean ± s.e.m. Student’s t-test. ****, P < 0.0001. B. Relative expression of itpl-1 gene in N. lugens in different tissues in the male reproductive system. Data are shown as means ± s.e.m. Groups that share at least one letter are statistically indistinguishable; Kruskal–Wallis test followed by Dunn’s multiple comparisons test with P < 0.05. C. The tissue distribution of itpl-1 analyzed by semi-quantitative RT-PCR. RNA samples from adult females, adult males, male accessary gland (AG) alone and adult male without accessary gland (male - AG). D-G. Relative expression of different spliceosomes of itpl-1–4 gene in males injected with dsRNA. Data are shown as mean ± s.e.m. Groups that share at least one letter are statistically indistinguishable; Kruskal–Wallis test followed by Dunn’s multiple comparisons test with P < 0.05. (TIF) [file pgen.1011699.s016.tif]

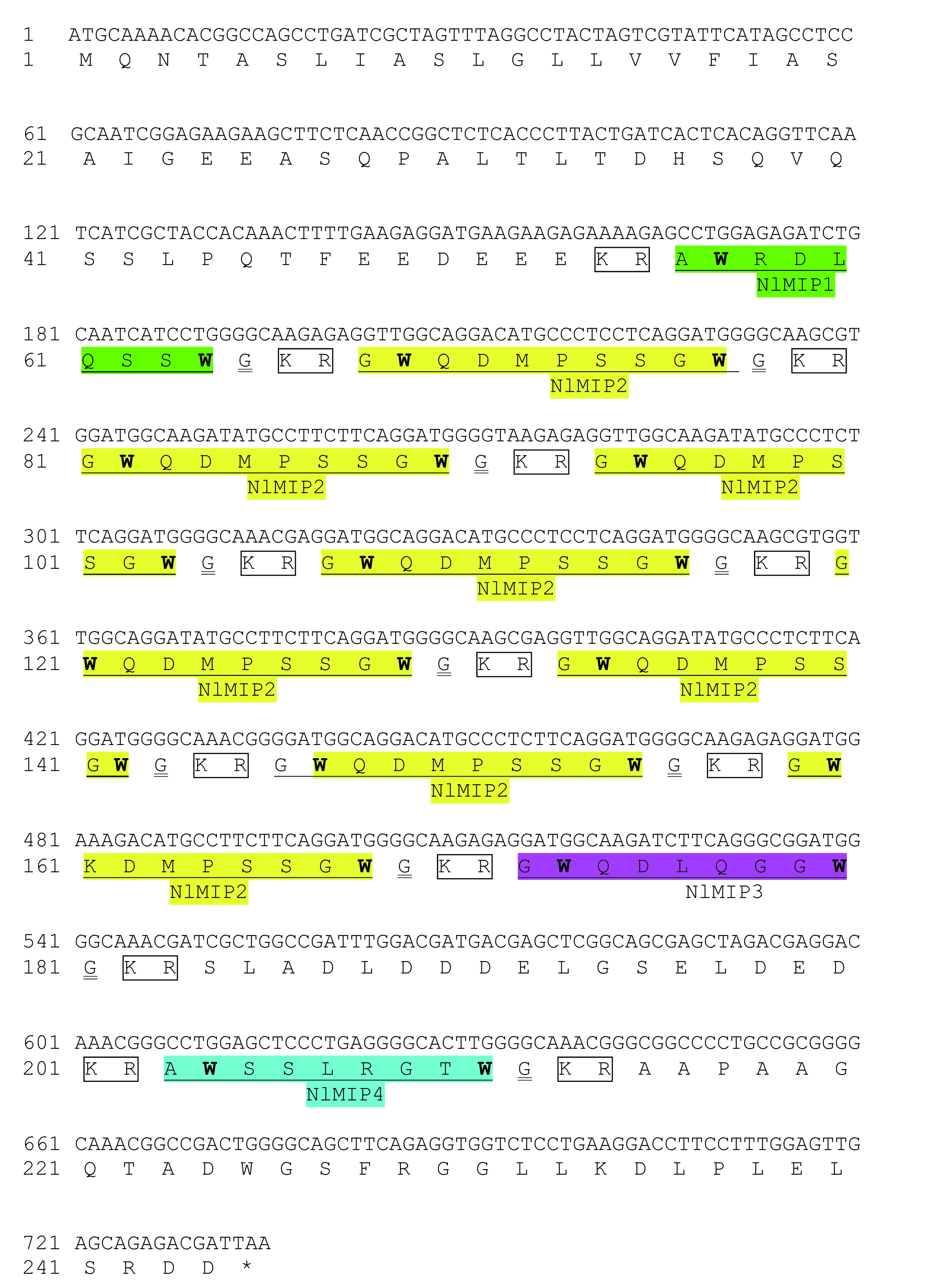

Supplement: S9 Fig — The four distinct mature peptides—MIP1 (green), MIP2 (yellow), MIP3 (purple), and MIP4 (cyan) are distinguished by unique colors. Cleavage sites (KR) are denoted by rectangular boxes, while the glycine residues (G) essential for amidation are highlighted by double underlining. (TIF) [file pgen.1011699.s017.tif]

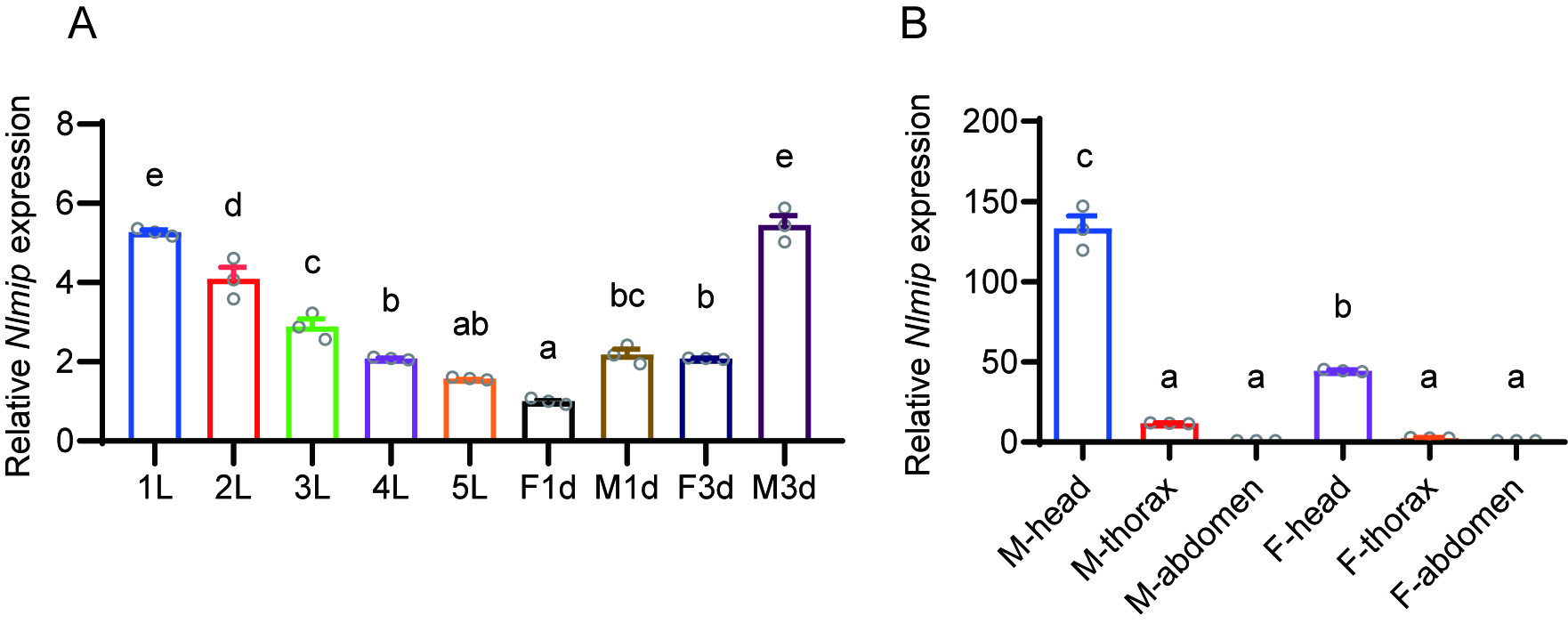

Supplement: S10 Fig — A. Relative expression of Nlmip gene in brown planthopper at different developmental stages. B. Relative expression of Nlmip gene in brown planthopper in different tissues. In the analysis of spatiotemporal expression patterns of mip, there were no less than 10 brown planthopper in each sample. M: male; F: female. Data are shown as means ± s.e.m. The presence of the same letter on the column indicates no significant difference between the two groups, and the absence of the same letter indicates a significant difference between the two groups. (TIF) [file pgen.1011699.s018.tif]

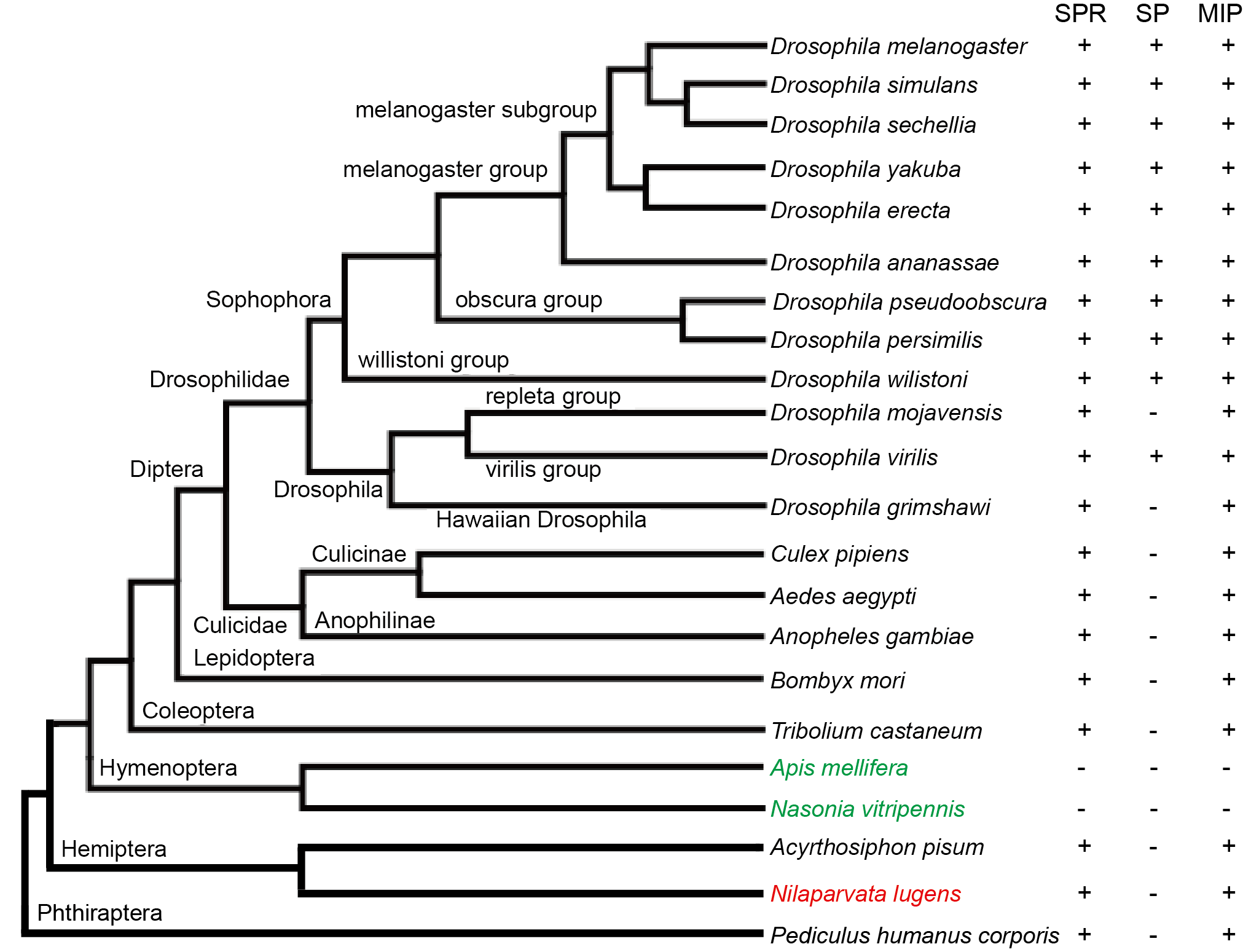

Supplement: S11 Fig — The brown planthopper (marked in red) lacks sex peptide. In Hymenopteran insects (marked in green), the honey bee Apis mellifera and the parasitic wasp Nasonia vitripennis, neither sex peptide, MIP, nor sex peptide receptor/MIPR are found. The “+” symbol represents presence, while the “-” symbol indicates absence. The phylogenetic tree of different insect species has been made by NCBI CommonTree https://www.ncbi.nlm.nih.gov/Taxonomy/CommonTree/wwwcmt.cgi. (TIF) [file pgen.1011699.s019.tif]

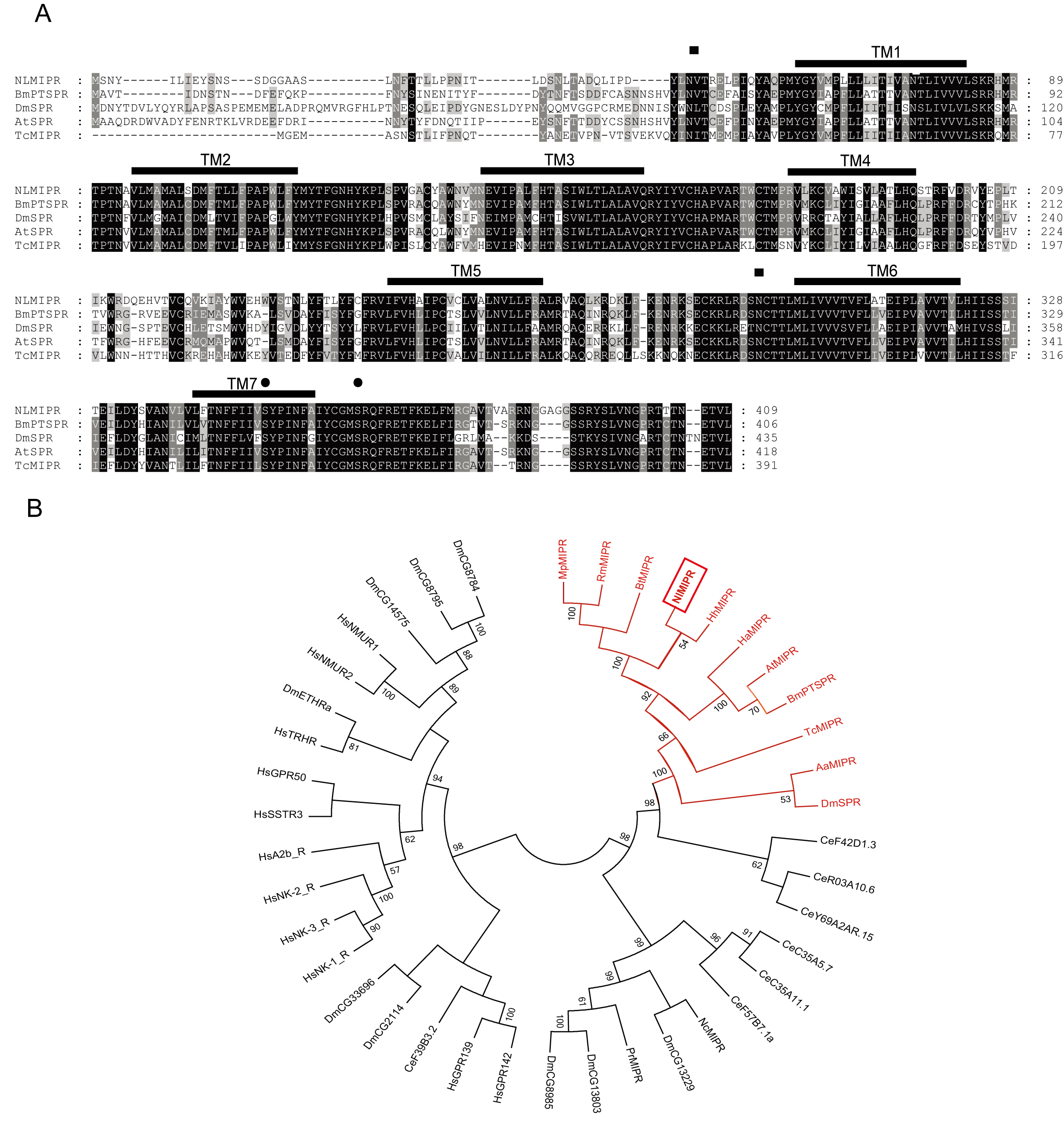

Supplement: S12 Fig — A Multiple comparison of MIPR in brown planthopper (NLMIPR) and four other insect species (Bombyx mori, Drosophila melanogaster, Amyelois transitella and Tribolium castaneum). The black lines (TM1-TM7) depict the transmembrane domains. B. Phylogenetic analysis of MIPRs in different insect species. (TIF) [file pgen.1011699.s020.tif]

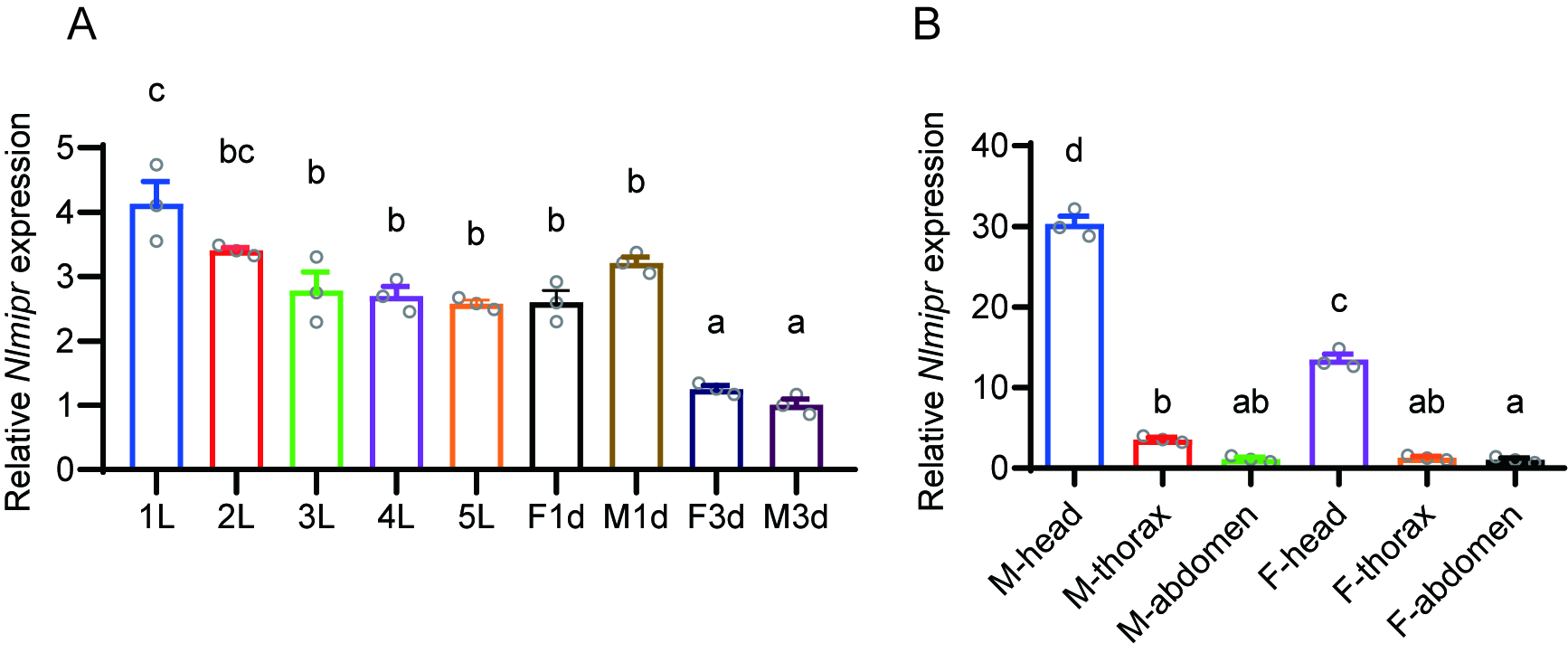

Supplement: S13 Fig — A. Relative expression of mipr gene in brown planthopper at different developmental stages. B. Relative expression of the mipr gene in different tissues of adult brown planthopper. M: male; F: female. Data are shown as means ± s.e.m. The presence of the same letter on the column indicates no significant difference between the two groups, and the absence of the same letter indicates a significant difference between the two groups. (TIF) [file pgen.1011699.s021.tif]
