# Supplementary material for: A novel male accessory gland peptide reduces female post-mating receptivity in the brown planthopper
Source: PLoS Genet. 2025 May 6;21(5):e1011699. doi: 10.1371/journal.pgen.1011699 (PMC12077777; doi:10.1371/journal.pgen.1011699)
Supplement: Attachment — Submitted filename: Response to Reviewers 3 Mar.docx [file pgen.1011699.s024.docx]

Dear Dr. Wu,

Thank you for submitting your manuscript to PLOS Genetics. After careful consideration, we feel that it has merit but does not fully meet PLOS Genetics's publication criteria as it currently stands. Therefore, we invite you to submit a revised version of the manuscript that addresses the points raised during the review process.

Please submit your revised manuscript within 60 days Feb 01 2025 11:59PM. If you will need more time than this to complete your revisions, please reply to this message or contact the journal office at plosgenetics@plos.org. When you're ready to submit your revision, log on to https://www.editorialmanager.com/pgenetics/ and select the 'Submissions Needing Revision' folder to locate your manuscript file.

Please include the following items when submitting your revised manuscript:

* A rebuttal letter that responds to each point raised by the editor and reviewer(s). You should upload this letter as a separate file labeled 'Response to Reviewers'. This file does not need to include responses to any formatting updates and technical items listed in the 'Journal Requirements' section below.

* A marked-up copy of your manuscript that highlights changes made to the original version. You should upload this as a separate file labeled 'Revised Manuscript with Track Changes'.

* An unmarked version of your revised paper without tracked changes. You should upload this as a separate file labeled 'Manuscript'.

If you would like to make changes to your financial disclosure, competing interests statement, or data availability statement, please make these updates within the submission form at the time of resubmission. Guidelines for resubmitting your figure files are available below the reviewer comments at the end of this letter.

We look forward to receiving your revised manuscript.

Kind regards,

Takaaki Daimon

Academic Editor

PLOS Genetics

Pablo Wappner

Section Editor

PLOS Genetics

Aimée Dudley

Editor-in-Chief

PLOS Genetics

Anne Goriely

Editor-in-Chief

PLOS Genetics

Additional Editor Comments:

The submitted manuscript has been evaluated by three reviewers.

As detailed below, all reviewers agreed that the manuscript presents significant advances in understanding post-mating responses in insects.

Therefore, I kindly request the authors to revise the manuscript accordingly.

Please note that Reviewer #2 has attached a file highlighting potential changes to the manuscript.

Response: Thank you for your letter and the reviewers’ comments on our manuscript. These comments are valuable and helpful. We have read the comments carefully and made suggested changes. According to the requirements of your letter, we have uploaded the revised manuscript file. Revisions in text are highlighted in red to show additions. Responses to reviewers are marked in red below. We would like to thank you for allowing us to resubmit a revised manuscript and we appreciate your time and consideration.

Reviewers' comments:

Reviewer's Responses to Questions

Comments to the Authors:

Please note here if the review is uploaded as an attachment.

Reviewer #1: The goal of this study was to characterize post mating responses in the brown planthopper, an important pest species, and determine the molecules involved in responses. Building on the existing body of knowledge regarding post-mating responses in Drosophila, they searched for a sex-peptide like molecule in the male brown planthopper seminal fluid that would induce post mating responses in females. They also characterized behavioral post mating stages in brown planthoppers and conducted a transcriptional and proteomic analysis of the male accessory gland fluid, followed by functional analysis of two candidate peptides (maccessin and ITPL-1). The authors ultimately determined that the newly discovered maccessin and ITPL-1 reduce female mating receptivity. No effects on the other post mating response tested, oviposition, were observed. The authors convincingly demonstrated effects of these peptides through transient knockdown with RNAi as well as injection of synthetic peptides. The authors then went on to investigate a receptor for maccessin, starting with an evaluation of myoinhibitory peptide receptors. They cloned the mip gene from the brown planthopper and characterized expression patterns and then injected the synthesized MIP peptides into females to no effect. Overall, this paper represents a vast source of new information on post mating responses in the brown plant hopper and should be of interest to researchers working on insect post mating responses. The manuscript could be improved in several ways including addition of significant methodological details, citations and statistical results.

Response: Thank you very much for your positive comments on our manuscript. According to your suggestions, we have revised the relevant parts of the manuscript. We respond to your queries point for point below.

Introduction:

LN 64. add “and others” to reflect the additional PMR phenotypes not listed here

Response: Corrected. This suggestion is appreciated (please see line 61).

LN 94. This statement is not accurate, the study by Duvall and others demonstrated that HP-1 has a short effect (less than 24hrs) on enforcing paternity. Other unknown factors control long-term refractory behavior.

Response: Sorry for this mistake and we are grateful for the suggestion. We have changed this statement as follows "interestingly HP-1 induces a short refractoriness (less than 24hrs) to insemination by other males. And other unknown factors control long-term refractory behavior." (please see line 90-93).

Results:

LN 162. The resistance to courting wears off after 24 hours in MAG-injected females. However, did you check all the time points in Fig.1A to be sure that there is no long-term effect of MAG injection?

Response: Thank you for your comment. We observed that 36 hours after injection, virgins injected with MAG protein showed no significant difference in mating acceptance rates compared with virgins injected with the control solvent. Since the receptivity rate of virgins after injection returned to normal levels at 36 hours, long-term mating receptivity rates after injection were not observed. We have also added these explanations to the manuscript (please see line 177-181)**.**

Fig.1. mean circles are hard to see (1B), what do the bars represent? I think SEM based on line 994, but this should be stated in the figure caption.

Response: Correct. We stated "Error bars indicate SEM throughout." in line 200 of the figure caption. We also made the mean bars larger than before (please see new Fig. 1).

170: Figure 1: more detail is needed on how categorization of females as either mated or virgin was. Confirmed. Did you check for presence of sperm in the spermatheca? Is courted a suitable proxy for mating?

Response: We selected fifth-instar nymphs from communal feeding boxes for solitary rearing. We collected newly emerged adult female brown planthoppers at 12-hour intervals for single-sex feeding. The reproductive system of the brown planthopper is not fully developed upon initial emergence, and the mating success rate within the first 24 hours post-emergence is less than 10% (Wang et al., 2020). Consequently, these early-emerged females were utilized as virgin test subjects for our experiments. These explanations can be found in lines 893 to 897 of the manuscript. Besides this, we stained the virgin and mated female copulatory bursa with DAPI (Figure 1 or new S2 Figure). Through microscopic observation, it was found that the copulatory bursa of the female after mating contains sperms transferred from the male (please see line 149-151).

Figure 1. Labeling of the sperm in the female copulatory bursa. The sperms were stained with DAPI. Scale bar: 100 μm.

Figure 1B: student’s t-test may not be the appropriate statistical test here since there are repeated measures.

Response: Thanks for your comment. We re-analyzed the data using two-way repeated measures ANOVA followed by holm-šídák's multiple comparisons test and found that there was a significant difference in the amounts of eggs laid by virgins and post-mating females at one to five days after mating. We also revised it in the figure legends (please see line 207).

LN 179-180. This statement needs a citation.

Response: This suggestion is appreciated. We have inserted the corresponding literature reference

(https://www.sciencedirect.com/science/article/pii/S0048357520300377) in LN 202-203.

LN 188-189. “The number of refractory females is high 3-6 h after SFP injection, but then declines.” How is this possible if in the methods (LN 344-345), the authors state that females need 6 hrs to recover from injections?

Response: Thank you for your comment. Our observations indicate that 6 hours after injection significantly affect the athletic ability of the females, while later times carry the potential risk of partial degradation of the injected substance by proteases within the females. Although the female's mating ability did not fully recover 6 hours after injection, it was still significantly different compared to the control group injected with the solvent. We can observe that 3-6 h after saline injection also decreases the receptivity of BPH with only 0.6 (See Fig. 1E of manuscript) (please see line 213-214).

LN 195. Fig. 1. Given the high variation in egg laying is this sample size (only 5 insects) adequate? Was a power analysis performed to determine the appropriate sample size? Is it appropriate to combine replicates? Show the statistical result demonstrating no replicate effect and that combining all samples was appropriate.

Response: This suggestion is appreciated. We are sorry for this mistake. Each replicate has at least ten insects. We have updated the presentation of Figure 1G. The egg laying experiment was performed in five biological replicates with no fewer than ten females per replicate. Five biological replicates were performed with at least ten insects per replicate for each experiment. See new figure legend (please see line 222-223).

LN 204. Indicate which is male and which is female (with an image, perhaps), for those less familiar with this insect.

Response: Thank you for the suggestion. The larger individual is the female and the smaller individual is the male. We have indicated this description to the figure. We've added the appropriate tags to the figure (please see new S1 Fig in Supporting Information file).

LN 219-220: What program was used to perform these QC steps?

Response: We used the NGSQC software to do these QC steps (Dai et al., 2010). Please see the line 235-236.

LN 223: Is there a reference you can cite for this genome?

Response: Done. We have added the reference here. Please see new line 240.

LN 229: A reference needed here

Response: We have added the reference here. We also described this method in the Materials and methods section. Please see new line 245-246.

LN 238: It is unclear what program was used for the GO terms analysis. More details are needed.

Response: We used GOseq (v1.10.0) for Gene ontology analysis for RNA-seq (Corrected P-Value<0.05) and InterProScan 5 to GO analysis for proteome. We have added the reference here. Please see new line 262-264.

LN 273 Fig. 2. This figure is difficult to interpret with so many categories. Consider combining categories. Perhaps follow convention used by other authors reporting similar data. One option would be to group all the go terms with less than e.g. 5 % and term them “other”. Otherwise, the color key is not particularly useful as there are many similar hues. All raw data should be made available to readers in the supplement or elsewhere.

Response: Thank you for the suggestion. We have merged the GO terms that account for less than 5%, and the new picture has been updated to Figure 2. The corresponding raw data can be downloaded from the China National Center for Bioinformation website (https://www.cncb.ac.cn/) by searching for the corresponding project number. Please see new line 962-977.

LN 276: Make the final sentence at the end of the figure legend more explanatory.

Response: Corrected. Please see new line 311-314.

There are no statistics in this GO term analysis. Are any of the GO terms significantly overrepresented in the data?

Response: Since we only sequenced the transcriptome and proteome of one sample, MAG, of *N. lugens*, we did not analyze the statistics in this GO term. We just analyze the major transcripts and proteins of MAG based on our omic’s data.

LN290 Fig. S2. What are the categories “overview” and “overview maps”?

Response: Overview is a second-level entry in the KEGG category, which is grouped under the first-level entry Metabolism and contains the following third-level entries: Carbon metabolism (ko01200), Biosynthesis of amino acids (ko01230), Fatty acid metabolism (ko01212) and 2-Oxocarboxylic acid metabolism (ko01210). Global and overview maps is also a second-level entry in the KEGG category, which is grouped under the first-level entry Metabolism and contains the third-level entries Metabolic pathways (ko01100). We have also added these explanations to the manuscript (please see line 294-305).

LN 302: In which tissue is the other variant of this gene produced in females?

Response: There are four transcripts of ITPL gene in brown planthopper. However, they encode the same mature ITPL peptide. Only the ITPL-1 transcript was expressed in MAG of male brown planthopper. The other three transcripts, ITPL-2, -3 and -4, were expressed both in males and females with much more expression in the integument (Yu et al., 2016b). *Itpl-2* and *itpl-3* are widely expressed in the integument, brain, the male or female reproductive system, gut (including Malpighian tubules) and fat body. *Itpl-4* is mostly expressed in the brain and integument (please see line 333-336).

LN 332: How many days post injection was the reduction in expression recorded? How long did the reduction in expression last?

Response: Gene expression levels were measured three days after injection (please see line 370). It was reported that dsRNA generally works on the genes of brown planthopper from one to seven days after injection. The detailed information can be found in the following article. Liu SH, Ding ZP, Zhang CW, Yang BJ, Liu ZW \(2010) Gene knockdown by intro-thoracic injection of double-stranded RNA in the brown planthopper, *Nilaparvata lugens*. Insect Biochemistry and Molecular Biology 40, 666-671. (Liu et al., 2010).

LN 305-306. More information on why these peptides were considered top candidates for further evaluation would be helpful. Were other molecules evaluated, and the data are just not shown?

Response: When we performed the function analysis, we found that the expression of this gene in the male accessory gland was very high, and it belonged to the signal transduction function in the classification. Amino acid sequence analysis revealed that its first 19 amino acids were predicted to be signal peptides. It may be a secreted protein that is more likely to be transferred to the female during mating. We have also added this explanation to the manuscript. (please see line 338-347). Besides, we selected several seminal fluid protein candidate genes with high expression levels for functional testing, and found that none of them had a significant effect on post-mating response (see Figure 2 in the figure below or new S5 Fig). And we have added these explanations to the manuscript. (please see line 338-347)

Figure 2. Receptivity of virgin and mated females after seminal fluid protein candidate genes were knocked down by dsRNA injections, scored as the percentage of females that copulated within 30 min. The different genes are referred to by their numbers. The small circles denote the number of replicates; the numbers below the bars denote total number of animals. Data are shown as mean ± s.e.m. **P* < 0.01, and ns (non-significant), *P* > 0.05, two-way repeated measures ANOVA followed by šídák's multiple comparisons test. The annotation of these seminal fluid genes were showed below:

Sfp52353: CLIP domain-containing serine protease 2. Sfp45778: melanization protease 1-like. Sfp48992: N(3)-methylcytidine methyltransferase METTL6. Sfp21953: GTP-binding nuclear protein Ran. Sfp21797: seminal fluid protein (APA33927.1). Sfp23008: seminal fluid protein (APA33928.1).

Figure 3A: The description of the different colors can go in the figure legend. It would be clearer just to show the amino acid sequence here.

Response: Thank you for the suggestion. We have made corrections in the figure and legend. Please see new Fig. 3A and legend.

Figure 3B: I assume the female used for RT-PCR was virgin, though it is unclear. This should be made clearer either in the figure or in the legend.

Response: We are grateful for the suggestion. We have added this information in the legend. Please see new Fig. 3B and legend.

Fig 3C: what time point post dsRNA injection were samples collected for qPCR? Is it 2 days post eclosion as this is when the egg laying assay was done? This needs to be clarified.

Response: This suggestion is appreciated. We injected dsRNA at day 3 of fifth instar nymph of brown planthopper. After 1 day, they will eclose. Two days after eclosion (three days after injection of dsRNA), the dsRNA-injection males were mated with virgin females (first mating). The mated females were tested for remating with another naïve male at one day later after first mating. The egg-laying assays were performed after the females had mated with dsRNA-injected males. We have added the description in the figure legend and also Methods part. Please see new Fig. 3C legend and line 863-869.

Fig 3D. “Such re-mating is never observed in the control (dsgfp-injected) group. However, the re-mating rate is small with only 7 percent of the females courted in the secondary mating being receptive” is this number statistically significant?

Response: Thanks for point out this. We also noticed the re-mating rate is small. However, this number is statistically significant. One reason might be that there is still Maccessin peptide produced since RNAi cannot completely eliminate this gene. The other reason is that there might be other molecules involved in the post mating response of brown planthopper. Please see new line 371-373.

Figure 3E/G: the markers on the graph denoting the mean are very hard to see. I don’t see that the standard error is shown on either graph, as suggested in the figure legend.

Response: We are grateful for the suggestion. We enlarged and bolded the average and SEM markers in the Figure 3E/G to make them more clearly visible. Please see new Fig 3E/G.

Figure 3 F/G/H: It would be clearer if the x axis labels were PBS-injected of Maccessin-injected. Otherwise, the difference between the dsRNA injections vs this new treatment is not clear.

Response: Thank you for the suggestion. We have corrected this in Figure 3. Please see new Fig 3F/G/H.

Figure 3H: if there were multiple replicates performed for this assay, you could show them on the graph, as is the case of other panels of the figure.

Response: We are grateful for the suggestion. We have also corrected this in Fig 3H.

Figure 3: In general, the positioning of the different elements of this figure are misaligned. Adjust position of graphs E, F and G and H so the X axes are in line with one another.

Response: This suggestion is appreciated. We have aligned these panels in Fig 3.

LN 356. Move information from A into the legend as this output is not necessary. It would be clearer just to show the amino acid sequence here.

Response: Thanks for your comment. We have removed the corresponding instructions from the figure and added them to the legend. Please see new Fig 3A.

LN 423-427. Was this result statistically significant?

Response: Yes, these results are statistically significant. We added data and difference analysis to figure and corresponding figure legends. Please see new line 458-461 and Fig 4A.

LN 445: Here, and elsewhere in the results section, statements are made about the data that are not accompanied by statistical tests. This needs to be addressed.

Response: We have added the statistical tests about the data in the figures and corresponding figure legends.

LN 503. Fig 4S. Gender is a social norm for humans. Sex is more appropriate here.

Response: Corrected. Please see new S8 Fig legend.

LN 535-539. In the expression analysis of mip at different life stages, it’s not clear in the text or in the graph how many biological replicates of this experiments were performed, nor how many individuals were used. There are no statistical tests performed here either to support the claims made that *mip* is more abundant in heads of males and females.

Response: Thanks for your comment. We have added the following information to the legend. In the analysis of spatiotemporal expression patterns of *mip*, there were no less than 10 brown planthoppers in each sample. The difference analysis of different groups has been marked in S10 Fig and figure legends. We have updated this figure in the revised paper. Please see the new supplementary materials file S10 Fig and line 542-546.

LN 559: Sometimes mipr is in caps/italics and sometimes not. This needs to be consistent.

Response: Thank you for the suggestion. MIPR caps means for proteins and *mipr* italics means for genes/transcripts. We make sure it is consistent in the manuscript.

LN 562-565: Satistics and/or sample sizes need to be included to support this statement.

Response: This suggestion is appreciated. The difference analysis of different groups has been marked in S13 Fig and figure legends. We have updated this figure in the revised paper. Please see the new supplemental materials file S13 Fig.

LN 607-608. Levels of kd can vary over time. More details on when kd was checked for these insects is needed; was kd level confirmed at the same day and time as the experiment was conducted. Figure 5G: please include in the figure legend or in the text at what point RNAi efficiency was measured. Was RNAi efficiency measured at the point of the receptivity assay and the remating assay? As I understand these assays were performed on different days. Do you know if the gene is still silenced at that point?

Response: We are grateful for the suggestion. The *dsgfp*- or *dsmipr*-injected females were collected immediately after the re-mating receptivity experiments were done. Hence, kd level was confirmed at the same day and time as the experiment was conducted. We make sure that the gene is still silenced when we performing behavioral experiments. We added these descriptions in the figure legend and methods part. Please see line 617-619.

Figure 5I: same comment as above about the size of the mean/s.e symbols.

Response: Thanks for your comment. We enlarged and bolded the average and SEM markers in the Figure 5I to make them more clearly visible. Please see the new Figure 5I.

Figure 5 supp2: In panel A there are no statistical tests to compare expression between life stages. It would be clearer to display the individual biological replicates on the graph here too, as in B. This also applies to Figure 5 supp 5 A.

Response: We are grateful for the suggestion. We have added the following information to the legend. The difference analysis of different groups has been marked in S10 Fig and figure legends. Please see the new supplementary materials file S10 Fig.

Discussion

LN 660. The opening sentence of the discussion is difficult to read. This should be split into two sentences.

Response: Thank you for the suggestion. We've broken that down to "Female behavior and physiological mating induction switches to ensure a large number of offspring, good viability, and ensure paternity. This behavior is common in insects, but only in fruit flies, where the underlying mechanism has been elucidated in detail, and the MAG-derived peptide SP has been identified as the primary secretory signal." Please see line 630-634.

LN 709. should read “was found to be affected”

Response: Thank you. Corrected. Please see line 682.

Methods

LN 770. Define IAM

Response: IAM stands for iodoacetamide. We have rewritten this part. Please see line 765-807.

LN 755. The description of MAG protein extraction needs more detail; to replicate this study it would be helpful for a reader to know centrifugation speeds and times, for example.

Response: Thank you for your comment. We have provided a more detailed description of the method for extracting male accessory gland proteins. After adding clean steel balls (Diameter 2mm) to the collected accessory gland samples, they were placed in a grinder and ground at 60 Hz for 2 minutes. After centrifugation at 10,000 g for 5 minutes, we collect the supernatant and washed the precipitate twice with 80% methanol. Please see line 734-745.

LN 846: Primer sequences do not appear to be included in the supplemental data and need to be made available. This applies also to the dsRNA primers referenced on line 885

Response: The primer sequence information is supplied in Supporting information Table S3. Please see new Table S3.

LN 966. Cite the reference for the Nilaparvata lugens genome

Response: Corrected. Please see line 759.

LN 973. A citation is needed for the DESeq2 R package

Response: Corrected. Please see line 762.

General comments and recommendations:

Present the statistical tests and results properly and consistently throughout.

Response: Corrected. We have added the statistical tests in the figures and corresponding figure legends.

More details on the methods are necessary in order to meet the rigor and reproducibility required by the journal.

Response: Corrected. We have added more detailed information in the methods part.

Use a consistent designation of the study organism. In some places BPH is used and in other *N. lugens* is used.

Response: We are grateful for the suggestion. We uniformly used *N. lugens* to refer to the insect in the manuscript.

Levels of kd can vary over time. More details on when kd was checked for these insects is needed; was kd level confirmed at the same day and time as the experiments were conducted?

Response: Yes, the kd level was confirmed at the same day and time as the experiments were conducted. We have described more clearly about this in this revised manuscript.

Reviewer #2: Dear editor and authors,

I have completed the review of the article entitled "A novel male accessory gland peptide reduces female post-mating receptivity in the brown planthopper”.

The work is well written and the data package is complete and solid. The authors address a very interesting topic from a theoretical and practical point of view. On the one hand, they describe a new protein found in the accessory glands of the males of a pest species, which inhibits the receptivity of the females after copulation but they go further, trying to elucidate the receptors that protein in the female, with sophisticated proteomic and transcriptomic methods.

From a practical point of view, the work is in a rice pest species; work aimed at the study of seminal fluids transferred from the male to the females during copulation and its effect on the behavior and physiology of the females are key to the future development of environmentally friendly control strategies.

I attach the MS with minimal suggestions and I encourage the authors to continue along this line of research aimed to elucidate the specific receptors in females on which the molecules that inhibit remating and trigger oviposition after copulation act, since these are topics studied almost exclusively in Drosophila and a bunch of insect species.

Response: Thank you for your positive and encouraging comments on our article. We plan to further investigate the post-mating response mechanism of the brown planthopper. According to your suggestions, we have corrected several mistakes in our original manuscript. Based on your comments, we also attached a point-by-point letter to you and the other two reviewers. We have made extensive revisions to our previous draft. The detailed point-by-point response is listed below.

LN138-140: Like in Tephritidae fruit flies (see papers of Eric Jang, Perez-Staples, Fritz, Abraham, Yuval, the Phil Taylor group, etc.).

Response: We are grateful for the suggestion. We have now cited he Tephritidae fruit flies references. Please see line 145.

LN149-152: Again, all the literature in tephritids was ignored, also in Callosobruchus, for example. May be you can find some interesting ones from a behaviural or physiological point of view:

Sirot L, Bansal R, Esquivel CJ, Arteaga-Vázquez M, Herrera-Cruz M, et al. 2021. Post-mating gene expression of Mexican fruit fly females: disentangling the effects of the male accessory glands. Insect Mol.Biol. 30(5):480–96

Yamane, T., Miyatake, T., Kimura, Y., 2008a. Female mating receptivity after injection of male-derived extracts in Callosobruchus maculatus. Journal of Insect Physiology 54, 1522-1527.

Yamane, T., Kimura, Y., Katsuhara, M., Miyatake, T., 2008b. Female mating receptivity inhibited by injection of male-derived extracts in Callosobruchus chinensis. Journal of Insect Physiology 54, 501-507.

Yamane, T., Miyatake, T., 2010. Induction of oviposition by injection of male-derived extracts in two Callosobruchus species. Journal of Insect Physiology 56, 1783-1788.

Abraham, S., Cladera, J., Goane, L. & Vera, M.T., 2012. Factors affecting Anastrepha fraterculus female receptivity modulation by accessory gland products. Journal of Insect Physiology, 58, 1-6.

Abraham, S., Nuñez-Beverido, N., Contreras-Navarro, Y. & Pérez-Staples, D., 2014. Female receptivity in Anastrepha ludens (Diptera: Tephritidae) is not modulated by male accessory gland products. Journal of Insect Physiology, 70, 41-48.

Brent ,C.S. & Hull, J.J., 2014. Characterization of male-derived factors inhibiting female sexual receptivity in Lygus hesperus. Journal of Insect Physiology, 60, 104-110.

Jang, E.B., 1995. Effects of mating and accessory gland injections on olfactory-mediated behavior in the female Mediterranean fruit fly, Ceratitis capitata. Journal of Insect Physiology, 41, 705-710.

Jang, E.B., McInnis, D.O., Kurashima, R., Carvalho, L.A., 1999. Behavioural switch of female Mediterranean fruit fly, Ceratitis capitata: mating and oviposition activity in outdoor field cages in Hawaii. Agricultural and Forest Entomology 1, 179-184.

Kuba, H. & Itô, I., 1993. Remating inhibition in the melon fly, Bactrocera (=Dacus) cucurbitae (Diptera: Tephritidae): copulation with spermless males inhibits female remating. Journal of Ethology, 11, 23-28.

Miyatake T., Chapman, T., Partridge, L., 1999. Mating-induced inhibition of remating in females Mediterranean fruit flies Ceratitis capitata. Journal of Insect Physiology 45, 1021-1028.

Mossinson, S., Yuval, B., 2003. Regulation of sexual receptivity of female Mediterranean fruit flies: old hypotheses revisited and new synthesis proposed. Journal of Insect Physiology 49, 561-567.

Radhakrishnan, P., Taylor, P.W., 2007. Seminal fluids mediate sexual receptivity and copula duration in Queensland fruit flies. Journal of Insect Physiology 53, 741-745.

Shutt, B., Stables, L., Aboagye-Antwi, F., Moran, J., Tripet, F., 2010. Male accessory gland proteins induce female monogamy in anopheline mosquitoes. Medical and Veterinary Entomology 24, 91-94.

Twig, E., Yuval, B., 2005. Function of multiple sperm organs in females Mediterranean fruit flies (Ceratitis capitata, Diptera: Tephritidae). Journal of Insect Physiology 51, 67-74.

Response: This suggestion is much appreciated. Thank you very much for the literature you recommended for us, which is very helpful to our understanding of post mating responses in different insect species. We have read and referred to this additional literature. Please see line 158-165.

LN155-156: only one spermatheca, right? Is there a ventral receptacle in the bursa copulatrix?

Response: Yes, there is only one spermatheca in the female reproductive system of brown planthopper. And there is no ventral receptacle in the bursa copulatrix of brown planthopper. The reproductive system morphology of brown planthopper and diptera insects is very different. Please see line 169-171.

LN168: Reyes-Hernández M, Córdova-García G, Díaz-Fleischer F, Flores-Estévez N, Pérez-Staples D. 2021.Oviposition after sex: mated Anastrepha ludens (Diptera: Tephritidae) females increase oviposition without receiving an ejaculate. Can. Entomol. 153(5):524–37

Response: we have inserted this reference here. Please see line 161-165.

LN528-529: Citation format error

Response: Corrected.

LN853: Citation format error

Response: Corrected.

LN892: What type of injector is used?

Response: Thank you for the query. We administered the injection using the WPI Micro4 Controller with a Micro Pump. We have added this information. Please see line 860-861.

The format of references (Latin scientific names are not italicized, the font size is inconsistent, and the case of letters is incorrect)

Response: We feel sorry for our carelessness. In our resubmitted manuscript, the typos have been corrected in the refereces part. Please see new reference list.

**Reviewer #3:** The manuscript by Zhang et al characterizes the female post-mating response (PMR) of Nilaparvata lugens in terms of female sexual receptivity and oviposition, as well as identifies a seminal fluid protein, macc, responsible for decreasing female sexual receptivity. The authors conducted an impressive amount of work, including RNAi-mediated gene silencing and injections of synthetic proteins into virgin females, to address the role of macc in modulating the PMR. They further examined the effects of ITPL-1 (another seminal fluid protein of N. lugens), the Sex Peptide of Drosophila melanogaster and the myoinhibitory peptide allatostatin-B (MIP) along with its receptor (MIPR).

The results are potentially intriguing for readers of PLOS Genetics as they provide new insights into molecular insect biology, reproductive physiology, and evolutionary genetics, fields of broad interest. However, the manuscript is significantly hindered by poor text organization, inadequate background information, insufficient justification of certain analyses, and numerous syntax and punctuation issues. In its current form, the writing is unacceptable for publication. Any revision should be thoroughly edited by someone fluent in English. Therefore, I will not list minor errors or issues but outline below some comments and suggestions for improvement.

Response: Thank you very much for your comments and professional advice. These points contribute to the academic rigor of our paper. According to your suggestions and requirements, we have made corrections to the revised manuscript. We hope the revised manuscript is acceptable for you. We address your queries and comments point for point below:

Introduction

1) Zhang et al. did not cite relevant studies that previously described the PMR in N. lugens. According to Yu et al. (2016; https://doi.org/10.1186/s12864-016-3013-7), mated females of this species exhibit stimulated oviposition levels (Long et al., 2010) and prolonged refractoriness to further insemination (Ichikawa, 1979). Although I could not access these articles, these findings indicate that the PMR has been studied before. The authors should incorporate this literature into their introduction to provide proper context and avoid presenting their results as if the PMR in N. lugens were previously uncharacterized.

Response: Thank you very much for your comments and advice. Long et al., did not study the egg laying behavior of virgin *N. lugens* (Long et al., 2010). They only studied the effects of delayed mating on reproduction in brachypterous females of the brown planthopper. Hence, their results did not provide direct evidence that mated females of *N. lugens* exhibit stimulated oviposition levels compared with unmated females (Long et al., 2010). Ichikawa et al., described some aspects of mating behavior of *N. lugens* (Ichikawa and Ishii, 1974). They found that female abdominal vibration will give males a signal for orientation. Neither of the two papers provides detailed information of mating and post mating behavior of BPH. Please see line 134-137.

2) The repertoire of seminal fluid proteins of *N. lugens* was already explored using transcriptomic and proteomic approaches (Yu et al., 2016). The authors should clarify why they conducted a new screen for male accessory gland (MAG) seminal fluid proteins.

Response: Thank you very much for your comment. Yes, Yu et al., have studied the repertoire of seminal fluid proteins of *N. lugens* using transcriptomic and proteomic approaches (Yu et al., 2016a). However, more novel sequencing technology has been developed since and the chromosome-level assembly of the brown planthopper genome with a characterized Y chromosome was reported in 2021 (Ye et al., 2021). Besides this, much more insect’s SFPs have been reported in recent years (Brown et al., 2023; Hurtado et al., 2022; Kerwin et al., 2020; Wigby et al., 2022). Hence, we conducted a new screen for male accessory gland (MAG) seminal fluid proteins using new sequencing technology and compared them to the new version genome of *N. lugens*. In our study, we found this orphan peptide (macc) which we performed functional studies of in this manuscript. Please see line 227-229.

3) Zhang et al. assert that macc is "the only candidate seminal fluid peptide that promotes a PMR in N. lugens." However, they also report similar effects for ITPL-1 on female receptivity. Since both proteins influence the PMR, it is incorrect to claim exclusivity for macc. Furthermore, other MAG-derived proteins may also play a role in the PMR. For instance, Ge et al. (2019) identified a MAG selenoprotein that affects oviposition levels in N. lugens. This relevant study is missing from the manuscript.

Response: Thank you very much for your comments and suggestions. We have changed our incorrect statement in the abstract of the manuscript. “Thus, macc is one of the important candidate seminal fluid peptides that inhibit receptivity of mated BPHs.” In addition, we have now described the work of Ge et al. (2019) in the revised manuscript introduction. Please see line 102-104.

4) The last paragraph of the introduction is overly focused on describing results and is therefore misplaced. It should be rewritten or moved to the Results section.

Response: Thank you for pointing this out. We have rewritten this part and shortened the last paragraph somewhat. Please see line 100-130.

5) The rationale for selecting macc as the focal protein is unclear. Was macc chosen because it was a novel seminal fluid protein, or were there other reasons? This should be explicitly explained to help readers understand the experimental framework. Additionally, the manuscript disproportionately emphasizes macc while treating ITPL-1 as secondary, even though both were studied using similar approaches and yielded comparable effects. The authors may consider separating the ITPL-1 findings into a distinct manuscript.

Response: Thanks for your comment. Importantly, unlike maccessin, ITPL-1 is produced also in females (and other tissues of males and females) and therefore it is likely that its main role is not as a seminal fluid peptide. It may also serve in the PMR of females as an endogenously released peptide (as we discuss). As for the emphasis on maccessin: it is indeed a novel peptide and we show that it is ONLY produced by the male accessory gland of male *N. lugens*. Therefore, it seems to play an exclusive role as a seminal fluid peptide. Furthermore, in the process of analyzing candidate seminal fluid protein genes, we found that macc was indeed specifically expressed in MAG and it has a signal peptide. This suggests that it is a secreted peptide that is likely to be transferred from males to females during copulation. Since its annotation in NCBI is ion-transport peptide precursor-like protein 1, we first started to analyze it as another analogue of ITPL, and found that its sequence is very different from ITPL. After verifying that its structure and distriubution not being ITPL-like, we renamed it as macc. We explain this in added text on line 116-121 and 338-347.

Results

6) The response variable used for characterizing female receptivity is unclear. Sometimes, the authors refer to the female willingness to accept courting males, giving the impression that they measured the female propensity to (re)mate, which is a reasonable proxy for receptivity. However, Fig. 1A seems to show the "Proportion of females courted", which is more related to attractiveness than to receptivity. Moreover, the Methods section vaguely describes the post-mating response assay: "The mated females were subjected to re-mating assay every 24 hours for 1–5 days after first mating, while the virgin females of the same age were used as controls." The age of the insects, experimental conditions, and specific response variables need clarification.

Response: Thank you for the suggestion. We are very sorry for the misunderstanding. Fig1A shows the willingness of females to accept courting males, rather than the attractiveness of females to males. Following your suggestions, a more detailed description of our behavioral experimental methods has been added to the manuscript. At the same time, the description of the test conditions is expanded. Hopefully, the improved descriptions of our experiments are satisfactory. Please see line 898-899.

Zhang et al. claim that MAG extracts demonstrate the role of seminal fluid proteins in the PMR. However, MAG extracts contain more than just seminal fluid proteins. This conclusion should be softened to acknowledge this limitation.

Response: We are grateful for the suggestion. We have changed the improper text on SFPs in the manuscript to MAG extracts. Please see new Fig 1.

8) The legends for some plots incorrectly state that "the small circles... denote total number of animals." Each circle likely represents an observation (e.g., the number of eggs laid by an individual female in Figure 1B). This needs correction.

Response: Thanks for your comment. We have corrected this in the legend of all the corresponding figure legends. Please see the new figure legend such as line 204.

9) Some legends are complete sentences (e.g., "The reproductive system of the male brown planthopper includes..."), while others are descriptive phrases (e.g., "Numbers of eggs laid per female"). The legends should consistently use concise, descriptive phrases.

Response: This suggestion is appreciated. We have changed all the sentences in the legend to concise and descriptive phrases. Please see the new figure legends.

10) Several methodological details currently appear in the Results section. For example: "Proteomic analysis was performed using Label-free... Production data was searched against brown planthopper MAG transcriptome databases using the Proteome Discoverer 2.2 (PD2.2, Thermo)." These details belong in the Methods section.

Response: Thank you for the suggestion. We have moved this description to the Methods section. Please see line 766-767 and 795-796.

11) The Results section mentions the use of Bowtie for read alignment, but the Methods section specifies HISAT2. Additionally, the Results refer to a de novo transcriptome assembly, which is not described in the Methods section. These inconsistencies need resolution.

Response: Thank you for pointing this out. We verified our experimental records and confirmed that the transcriptome sequences were spliced using Trinity (v2.4.0) software. The parameter is min_kmer_cov:3, and other parameters are default. We redescribe the transcriptome section of the material approach in more detail. Please see line 746-764.

12) The subsection "Transcriptome and proteome analysis of male accessory glands" is confusing and incomplete. Key data points, such as the number of transcripts/genes transcribed in the MAGs and the proportion translated into proteins, are missing. After revision, this content may fit better within the "Identification of seminal fluid proteins" subsection.

Response: Thank you for your comment. In response to your question, we have added descriptions of the corresponding data of the transcriptome and proteome of the manuscript. Please see line 252-261.

13) The criteria for predicting seminal fluid proteins from the data are missing. Additionally, there is no comparison between the proteins identified in this study and those reported by Yu et al. (2016). This comparison would strengthen the manuscript.

Response: We are grateful for the suggestion. In response to your question, we have added the following description in the manuscript.

“Genes encoding SFPs were predicted using both the N. lugens transcriptome assembly and proteomic analysis. The screening criteria for seminal fluid proteins (SFPs) in the brown planthopper (N. lugens) are as follows:

1. Gene Annotation: Genes labeled as "seminal fluid protein" in previous studies were selected through screening gene annotations.

2. Gene Expression Level: The gene expression levels in the MAG (metagenome - assembled genome) were recorded and ranked from high to low.

3. Signal Peptide Presence: Genes were further checked for the presence of signal peptides.

4. Candidate Selection: Genes that have high expression levels in the MAG and also possess signal peptides, among the "seminal fluid protein" genes, are considered as candidate genes for SFPs.” Please see line 317-328.

14) The authors conclude that "neither MIP nor MIPR is required for increased post-mating oviposition." While this is likely, the lack of gene knockout experiments weakens this conclusion. It should be softened accordingly.

Response: This suggestion is appreciated. We have softened our statement in the manuscript. Please see line 589-591.

Discussion

15) Apart from SP and DUP99, other influential seminal fluid proteins of *D. melanogaster* (e.g., ovulin) should be included in the discussion to provide broader context.

Response: This suggestion is appreciated. We have added the description of ovulin and the associated reference in the discussion part. Please see line 676-683.

M&M

16) The Methods section is incomplete, making it impossible to replicate the experiments or analyses. Essential details must be included.

Response: Thank you for pointing this out. We have carefully checked the manuscript and added information. Please see our revised methods part.

17) The Methods subsections should be reordered to match the sequence in which the results are presented. For instance, placing the "Behavior Assays" subsection between "Mass Spectrometry" and "Proteome Analysis" is confusing and should be changed.

Response: Thank you for your comments. We have reordered the Methods subsections to match the sequence of presented results and placed sections "RNA-seq analysis" and "Proteome analysis" immediately after "Extraction of male accessory glands proteins".

References

Brown, N.C., Gordon, B., McDonough-Goldstein, C.E., Misra, S., Findlay, G.D., Clark, A.G., Wolfner, M.F., 2023. The seminal odorant binding protein Obp56g is required for mating plug formation and male fertility in Drosophila melanogaster. eLife 12, e86409.

Dai, M., Thompson, R.C., Maher, C., Contreras-Galindo, R., Kaplan, M.H., Markovitz, D.M., Omenn, G., Meng, F., 2010. NGSQC: cross-platform quality analysis pipeline for deep sequencing data. BMC Genomics 11, S7.

Hurtado, J., Almeida, F.C., Belliard, S.A., Revale, S., Hasson, E., 2022. Research gaps and new insights in the evolution of Drosophila seminal fluid proteins. Insect Molecular Biology 31, 139-158.

Ichikawa, T., Ishii, S., 1974. Mating Signal of the Brown Planthopper, Nilaparvata lugens ST&Aring;L (Homoptera : Delphacidae) : Vibration of the Substrate. Applied Entomology and Zoology 9, 196-198.

Kerwin, P., Yuan, J., von Philipsborn, A.C., 2020. Female copulation song is modulated by seminal fluid. Nature Communications 11, 1430.

Liu, S., Ding, Z., Zhang, C., Yang, B., Liu, Z., 2010. Gene knockdown by intro-thoracic injection of double-stranded RNA in the brown planthopper, Nilaparvata lugens. Insect Biochem. Mol. Biol. 40, 666-671.

Long, Y., Hou, M.-L., Yang, X., Shi, B.-K., 2010. Effects of delayed mating on reproduction in brachypterous females of the brown planthopper, *Nilaparvata lugens*. Plant Protection 36, 36-39.

Wang, L.X., Zhang, Y.C., Tao, S., Guo, D., Zhang, Y., Jia, Y.L., Zhang, S., Zheng, C., Khan, D., Gao, C.F., Wu, S.F., 2020. Pymetrozine inhibits reproductive behavior of brown planthopper Nilaparvata lugens and fruit fly Drosophila melanogaster. Pestic Biochem Phys 165.

Wigby, S., Brown, N.C., Sepil, I., Wolfner, M.F., 2022. On how to identify a seminal fluid protein: A commentary on Hurtado et al. Insect Molecular Biology 31, 533-536.

Ye, Y.-X., Zhang, H.-H., Li, D.-T., Zhuo, J.-C., Shen, Y., Hu, Q.-L., Zhang, C.-X., 2021. Chromosome-level assembly of the brown planthopper genome with a characterized Y chromosome. Molecular Ecology Resources n/a.

Yu, B., Li, D.-T., Lu, J.-B., Zhang, W.-X., Zhang, C.-X., 2016a. Seminal fluid protein genes of the brown planthopper, *Nilaparvata lugens*. BMC Genomics 17, 654.

Yu, B., Li, D.T., Wang, S.L., Xu, H.J., Bao, Y.Y., Zhang, C.X., 2016b. Ion transport peptide (ITP) regulates wing expansion and cuticle melanism in the brown planthopper, *Nilaparvata lugens*. Insect Molecular Biology 25, 778-787.
