# Supplementary material for: A novel male accessory gland peptide reduces female post-mating receptivity in the brown planthopper
Source: PLoS Genet. 2025 May 6;21(5):e1011699. doi: 10.1371/journal.pgen.1011699 (PMC12077777; doi:10.1371/journal.pgen.1011699)
Supplement: Attachment — Submitted filename: second response letter-4.10.docx [file pgen.1011699.s025.docx]

PGENETICS-D-24-01238R1

A novel male accessory gland peptide reduces female post-mating receptivity in the brown planthopper

PLOS Genetics

Dear Dr. Wu,

Thank you for submitting your manuscript to PLOS Genetics. After careful consideration, we feel that it has merit but does not fully meet PLOS Genetics's publication criteria as it currently stands. Therefore, we invite you to submit a revised version of the manuscript that addresses the points raised during the review process.

Please submit your revised manuscript within 30 days May 02 2025 11:59PM. If you will need more time than this to complete your revisions, please reply to this message or contact the journal office at plosgenetics@plos.org. When you're ready to submit your revision, log on to https://www.editorialmanager.com/pgenetics/ and select the 'Submissions Needing Revision' folder to locate your manuscript file.

Please include the following items when submitting your revised manuscript:

* A rebuttal letter that responds to each point raised by the editor and reviewer(s). You should upload this letter as a separate file labeled 'Response to Reviewers'. This file does not need to include responses to formatting updates and technical items listed in the 'Journal Requirements' section below.

* A marked-up copy of your manuscript that highlights changes made to the original version. You should upload this as a separate file labeled 'Revised Manuscript with Track Changes'.

* An unmarked version of your revised paper without tracked changes. You should upload this as a separate file labeled 'Manuscript'.

If you would like to make changes to your financial disclosure, competing interests statement, or data availability statement, please make these updates within the submission form at the time of resubmission. Guidelines for resubmitting your figure files are available below the reviewer comments at the end of this letter.

We look forward to receiving your revised manuscript.

Kind regards,

Takaaki Daimon

Academic Editor

PLOS Genetics

Pablo Wappner

Section Editor

PLOS Genetics

Aimée Dudley

Editor-in-Chief

PLOS Genetics

Anne Goriely

Editor-in-Chief

PLOS Genetics

Additional Editor Comments:

This revised manuscript has been evaluated by original reviewers.

In light of their comments, I would like to request a final round of revision.

As pointed out by Reviewer #3, please reduce the use of abbreviations and prioritize those that appear frequently to improve the readability of the manuscript.

Response: We sincerely appreciate the reviewers' constructive feedback and have carefully addressed all remaining concerns. We have significantly reduced non-essential abbreviations throughout the manuscript, retaining only those that appear ≥10 times in the text or are essential for technical descriptions. All abbreviations are now properly defined at first mention.

Additionally, numerical data that underlies graphs or summary statistics should be provided in spreadsheet form as supporting information. Please submit a new supplementary table that includes numerical data for Figs. 1, 3, 4, 5, S5, S8, S10, and S13.

Response: We have created a comprehensive supplementary table (Supplementary Table S5) containing all raw numerical data for Figs. 1, 3, 4, 5, S5, S8, S10, and S13. We believe these revisions substantially improve manuscript clarity and data transparency. Thank you for the opportunity to strengthen our work. Please find below our point-by-point responses.

Journal Requirements:

Please ensure that the funders and grant numbers match between the Financial Disclosure field and the Funding Information tab in your submission form. Note that the funders must be provided in the same order in both places as well.

Response: Corrected.

Reviewers' comments:

Reviewer's Responses to Questions

Comments to the Authors:

Please note here if the review is uploaded as an attachment.

Reviewer #2: Dear authors and editor,

I have just finished reading the other reviewers' corrections, almost all of which have been adopted by the authors, including my own. Approximately 25 articles that should be cited have been added, and the materials and methods have been considerably expanded to make the methodologies reproducible.

The authors have done extensive work improving the manuscript, taking into account the considerations of the three reviewers. Therefore, I consider it worthy of publication.

Response: We are deeply grateful for your positive assessment of our revisions. Thank you for acknowledging our efforts to our study. Your constructive feedback during the review process has significantly improved the quality of this work. We appreciate your final endorsement for publication.

Reviewer #3: I thoroughly enjoyed reading this interesting manuscript. The authors have done an excellent job addressing the reviewers' comments from the initial review. I now have no major concerns about the quality of this manuscript and am happy to recommend it for publication. However, I do have some minor comments that should be straightforward to address:

Response: We sincerely appreciate your positive evaluation and recommendation for publication. Thank you for recognizing our efforts in addressing the previous concerns comprehensively. We have carefully considered all remaining minor comments and provide detailed responses below.

1. Abbreviations: The manuscript currently contains a large number of abbreviations, which can make it difficult for readers to follow. I suggest either reducing the number of abbreviations or including a table of abbreviations for reference. Below is a list of the abbreviations used throughout the manuscript:

PMR: post-mating response

SP: sex peptide

MAG: male accessory glands

macc: maccessin

SPR: sex peptide receptor

MIP: myoinhibitory peptide

MIPR: myoinhibitory peptide receptor

HP-1: head peptide

SPF-L: Selenoprotein F-Like

ITPL-1: ion transport peptide-like

SFP: seminal fluid protein

ITPa: recombinant amidated ITP

BPH: brown planthopper

sNPF: short neuropeptide F

NPYLR1: short neuropeptide F receptor

Response: We appreciate your constructive feedback regarding abbreviation usage. In accordance with your recommendation, we have eliminated nine non-essential abbreviations from the original list, retaining only those meeting frequency criteria (appearing ≥ 10 times) and technical necessity: PMR, MAG, macc, MIP, MIPR, ITPL-1 and ITPa. Each retained abbreviation is now consistently defined at first mention.

2. Line 188: Please add "the" before "Mexican fruit fly" for grammatical correctness.

Response: Corrected.

3. Line 189: Replace "we" with "our findings" or "our results".

Response: Corrected.

4. Figures 1A, 4A, 5A, and S5A: The vertical axis title "Proportion of females courted" could be misleading, as it may imply that female attractiveness was assessed rather than receptivity. Consider revising it to "Proportion of females accepting courting males" or something similar to clarify the intended meaning.

Response: Thanks for the suggestion. We refer to the article that revealed the mechanism of post-mating response in *Drosophila melanogaster*, and change the vertical axis title to "Copulation within 30 min (%)".

5. Line 226: Add "brown planthopper" before "(BPH)" because the abbreviation was first introduced here.

Response: Thank you for your advice. We have changed all the “BPH” abbreviations in the manuscript to “brown planthopper”.

6. Lines 233–244: To improve flow and clarity, consider explicitly stating whether a de novo or genome-guided assembly was performed before describing the sequence similarity searches of the assembled transcripts. This would help connect the pre- and post-assembly parts of this paragraph.

Response: Thank you for this insightful suggestion. We have revised line 234-235 to explicitly clarify the assembly strategy. A de novo transcriptome assembly was performed using Trinity v2.4.0 (min_kmer_cov:3, group_pairs_distance 500).

7. Lines 329–330: The numbers 373 and 209 do not match the values shown in Figure S3B (394 and 205, respectively). Please revise these numbers for accuracy.

Response: Thank you for identifying this discrepancy. The inconsistency resulted from an outdated version of Figure S3B that was not updated during the final revision. The numbers 373 and 209 are correct. We have now rigorously addressed this problem. We apologize for this oversight and appreciate your meticulous review, which has improved the manuscript's technical precision.

8. Lines 331–332: The SignalP column is missing from Table S2. Please ensure this column is included to provide complete information.

Response: We are grateful for the suggestion. We have implemented this suggestion as requested. The SignalP prediction column has now been added to Table S2. This addition ensures completeness of the bioinformatics analysis metadata.

9. Lines 340–342: In addition to macc, Table S2 includes nearly a dozen other unknown SFP genes with higher expression levels in the MAGs and detected peptide fragments. It would be helpful to provide a more detailed explanation of why macc was chosen for further exploration over these other genes. For example, was it selected because it was the only one with a predicted signal peptide, or due to its annotation as an ion transport peptide precursor? Also, please clarify whether macc was included in the list of putative seminal fluid proteins or seminal fluid protein candidates.

Response: Thank you for raising this important clarification. According to the results of gene annotation and analysis, we believe that the candidate genes of SFP with signal transduction function may play a more important role. Among these candidate genes, the expression of itpl and macc annotated as an ion transport peptide precursor is very high and there are predicted signal peptides. Macc is included in the candidate genes of seminal fluid protein. We have added the following explanations to Lines 341-351.

10. Line 756: Please clarify the type of assembly performed using Trinity software (e.g., de novo or genome-guided) and include relevant run parameters to provide a complete description of the methods.

Response: This suggestion is appreciated. A de novo transcriptome assembly was performed using Trinity v2.4.0 (min_kmer_cov:3, group_pairs_distance 500). We have added this to lines 234-235.

Have all data underlying the figures and results presented in the manuscript been provided?

Large-scale datasets should be made available via a public repository as described in the PLOS Genetics data availability policy, and numerical data that underlies graphs or summary statistics should be provided in spreadsheet form as supporting information.

Reviewer #2: Yes

Reviewer #3: No: It may not be necessary, but some data e.g., underlying bar plots were not provided as supporting information tables.

Response: We have created a comprehensive supplementary table (Supplementary Table S5) containing all raw numerical data for Figs. 1, 3, 4, 5, S5, S8, S10, and S13. We believe these revisions substantially improve manuscript clarity and data transparency. Thank you for the opportunity to strengthen our work.
